# Transcriptomic Profiling Unravels the Disruption of Photosynthesis Apparatuses and Induction of Immune Responses by a Bipartite Begomovirus in Tomato Plants

**DOI:** 10.3390/plants13223198

**Published:** 2024-11-14

**Authors:** Wen-Ze He, Ting Rong, Xun-Yue Liu, Qiong Rao

**Affiliations:** Zhejiang Key Laboratory of Biology and Ecological Regulation of Crop Pathogens and Insects, College of Advanced Agricultural Sciences, Zhejiang A&F University, Hangzhou 311300, China; hewenze@zafu.edu.cn (W.-Z.H.); rongting@stu.zafu.edu.cn (T.R.); 20150008@zafu.edu.cn (X.-Y.L.)

**Keywords:** tomato yellow leaf curl disease, tomato yellow leaf curl Thailand virus, *Solanum lycopersicum*, transcriptome, photosynthesis, plant antiviral defense

## Abstract

Diseases caused by begomoviruses such as tomato yellow leaf curl disease (TYLCD) are major constraints in agriculture. While the interactions between plants and monopartite begomoviruses during TYLCD pathogenesis have been explored extensively, how bipartite begomoviruses interact with tomato plants are understudied. Here we first found that a bipartite begomovirus tomato yellow leaf curl Thailand virus (TYLCTHV) induced stunted growth, leaf curl and yellowing in tomato plants. We then profiled the tomato transcriptomic changes in response to TYLCTHV infection. In total, we identified 2322 upregulated and 1377 downregulated genes. KEGG enrichment analysis of the differentially expressed genes (DEGs) revealed that many KEGG pathways regulating plant photosynthesis processes and defenses were enriched. Specifically, TYLCTHV infection disrupted the expression of DEGs that function in the light-harvesting chlorophyll protein complex, photosystem I and II, cytochrome b6/f complex, photosynthetic electron transport and F-type ATPase. Additionally, the expression of many DEGs regulating plant defenses including pathogen-associated molecular pattern (PAMP)-triggered immunity, effector-triggered immunity and hypersensitive response was upregulated upon TYLCTHV infection. Taken together, we found that during the pathogenesis of TYLCD induced by TYLCTHV, the virus actively disrupts plant photosynthesis processes and induces defense responses. Our findings add to our knowledge of TYLCD pathogenesis and plant–virus interactions in general.

## 1. Introduction

Plants are often attacked by many pests including pathogens and herbivores in both natural and agricultural ecosystems. Of these pathogens, the non-cellular infectious agents viruses are obligate parasites that exclusively live and multiply within their plant hosts [1]. In agriculture, viral pathogens cause substantial losses in the yield and quality of many crops [2]. For example, all the major staple crops including rice, maize and wheat are threatened by viral pathogens in many grain production areas [2]. In recent decades, diseases caused by whitefly-borne begomoviruses (family *Geminiviridae*) have substantially affected the production of many important crops in various regions worldwide [3]. Diseases caused by begomoviruses include cassava mosaic, cotton leaf curl and tomato yellow leaf curl, among others [3]. While some control methods are available, limited success has been achieved for the majority of plant viruses, highlighting the urgent need to disentangle viral pathogenesis and epidemics and in turn develop novel control strategies [2].

Upon the invasion of viral pathogens, intimate interactions may occur between these obligate parasites and their plant hosts [4]. As viruses rely on host machineries for multiplication, viruses often significantly reprogram plant processes so as to create a micro-environment conducive to virus replication [4,5]. For example, geminiviruses reprogram plant cell cycle and developmental controls, leading to the increased availability of host replisomes that are required for geminivirus multiplication [5]. Additionally, extensive virus replication within plant cells may alter the structure and/or function of organelles such as chloroplasts, leading to the disruption of cellular processes in infected plants [4]. Plants, on the other hand, are equipped with a battery of defenses to fend off viral pathogens, including gene silencing and phytohormonal pathways such as salicylic acid [6]. To sustain their persistence within plant hosts, viruses have evolved an array of proteins that actively suppress plant antiviral immunity [6]. Dissecting these intimate molecular interplays is instrumental in understanding viral pathogenesis and the improvement of plant resistance to these notorious pathogens.

Tomato yellow leaf curl disease (TYLCD) represents one of the most significant diseases in the production of tomato (*Solanum lycopersicum*) worldwide [7]. The symptoms of TYLCD include the yellowing and curling of leaves, stunted growth and a dramatically reduced fruit yield [7]. TYLCD can be caused by many begomoviruses, including monopartite ones such as tomato yellow leaf curl virus and bipartite ones such as tomato yellow leaf curl Thailand virus (TYLCTHV). While the genome of monopartite begomoviruses contains only one single-stand circular DNA, that of bipartite begomoviruses consists of two DNA molecules with one (DNA-A) resembling the genome of monopartite begomoviruses [8]. The outbreaks of TYLCD in the last decades have sparked many investigations on the characterization of causal agents and dissection of disease pathogenesis [7]. Notably, however, while the monopartite begomovirus tomato yellow leaf curl virus has been studied extensively in many aspects of biology, bipartite begomoviruses including TYLCTHV are understudied [7,9]. First isolated from TYLCD-infected tomato plants in Thailand, TYLCTHV is a typical bipartite begomovirus that is transmitted by the whitefly *Bemisia tabaci* in a persistent-circulative mode [10,11,12,13]. During the last decades, TYLCTHV has gradually spread from its Thailand origin to neighboring tomato-growing countries [14]. Nowadays, TYLCTHV has become widespread in East and Southeast Asian regions such as Myanmar, Indonesia, Thailand and Taiwan province of China [14,15,16,17,18].

Considering the knowledge gaps in TYLCTHV biology, here we probed plant-TYLCTHV interactions by profiling the transcriptomic responses of tomato plants to TYLCTHV infection. We first examined the effects of TYLCTHV on tomato plant phenotype. Next, we profiled the transcriptomic landscape of tomato plants in response to TYLCTHV infection. Finally, we analyzed differentially expressed genes so as to unveil the modulation of tomato plant biological processes by TYLCTHV. The findings in this study will contribute to an improved understanding of TYLCD pathogenesis and begomovirus–plant interactions.

## 2. Results

### 2.1. Phenotypes of Control and TYLCTHV-Infected Plants

**Figure 1:**
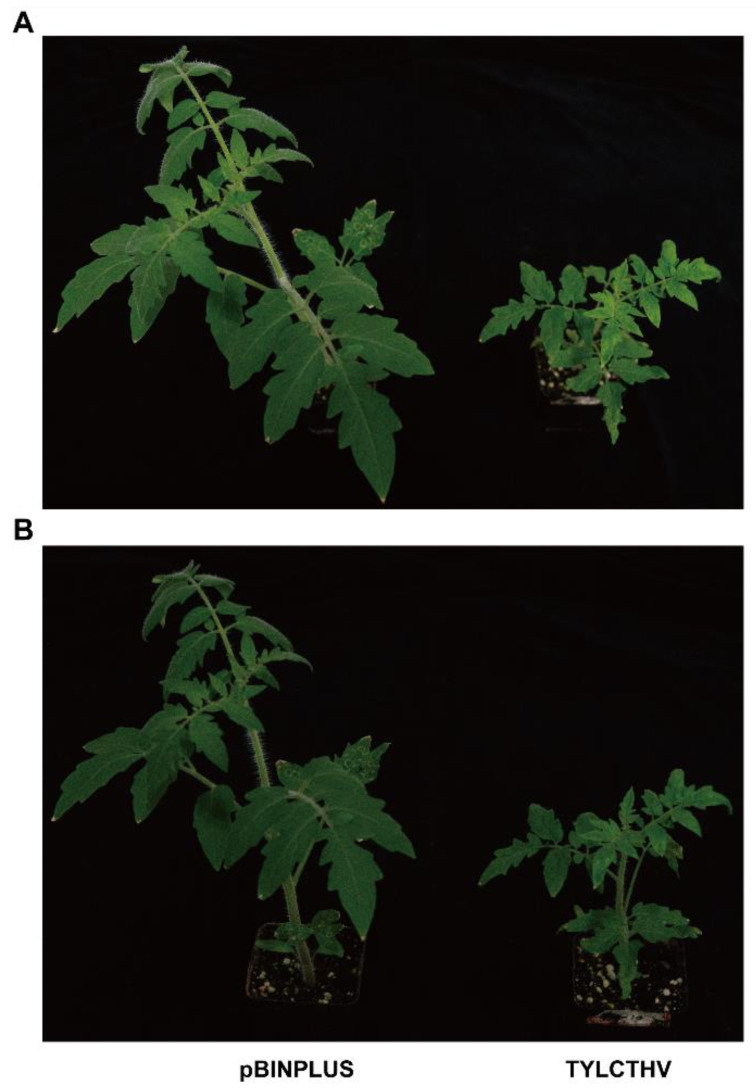
Symptoms induced by TYLCTHV in tomato plants. Tomato plants were inoculated with pBINPLUS (empty vector) and TYLCTHV DNA-A + DNA-B. (**A**) Top view of pBINPLUS-inoculated and TYLCTHV-inoculated tomato plants. (**B**) Side view of tomato plants. Pictures were taken at three weeks post-inoculation.

Pictures of tomato plants at three weeks post-inoculation are presented in Figure 1. Notably, when compared to control, the leaves from TYLCTHV-infected tomato plants were much smaller (Figure 1A). Additionally, downward leaf curl and yellowing were found in the young leaves of tomato plants (Figure 1A). When examining the whole plants, the control plants were substantially taller than infected plants (Figure 1B). PCR verified the absence and presence of TYLCTHV DNA-As in pBINPLUS- and TYLCTHV-inoculated plants (Figure S1). Based on the results of symptom inspection and PCR detection, we chose tomato plants at three weeks post-inoculation for subsequent experimentation.

### 2.2. RNA-Seq and Genome Mapping

Four samples were analyzed for both control and TYLCTHV-infected plants. For each sample, 43,688,846–64,534,292 raw reads were obtained; after filtering, 43,341,960–63,907,418 clean reads were obtained (Table 1). The sequencing error rates were 0.0122–0.0124%, and the Q20 and Q30 values were over 98% and 95%; the GC contents were 41.64–43.42% (Table 1). Mapping to the reference tomato genome was then conducted for the clean reads. The total mapping rate was about 94.55–98.23% (Table 2). The rates of multiple mapped were below 4.07%, and the unique mapped rates were 90.79–95.00% (Table 2). Based on the *Solanum_lycopersicum* reference genome (SL4.0, https://www.solgenomics.net/organism/Solanum_lycopersicum/genome/, accessed on 8 October 2024), the aligned reads were assembled. In total, 51,909 transcripts were obtained. The majority (64.9%) of the transcripts were over 1000 bp and only 2.37% of transcripts were shorter than 200 bp (Figure S2). 

### 2.3. Analysis and Functional Annotation of Differentially Expressed Genes (DEGs)

**Figure 2:**
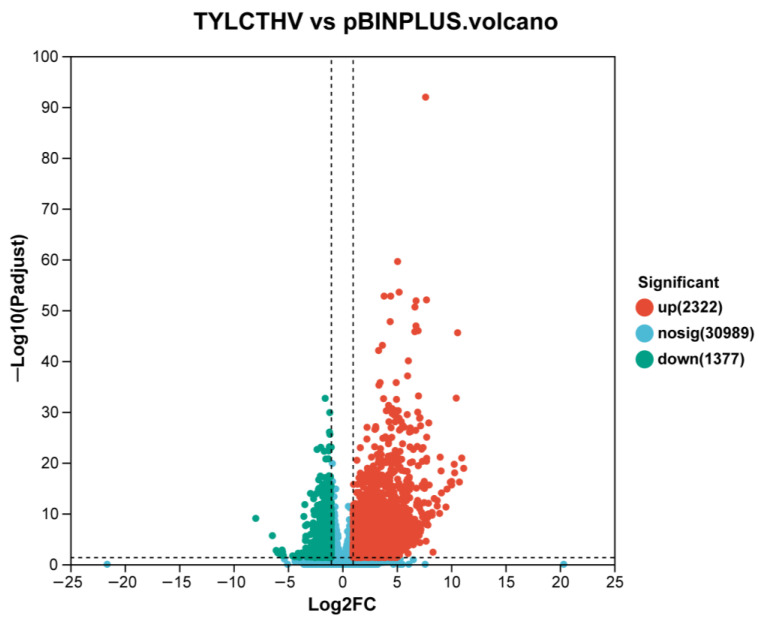
Volcano plot of the gene expression profile upon TYLCTHV infection. Each point in the plot represents a specific gene. Red points indicate significantly upregulated genes, blue points represent significantly downregulated genes and gray points represent genes whose expression exhibits no significant difference (nosig) between the two treatments. The numbers of genes in each category are presented in the plot.

The expression level of all genes in TYLCTHV-infected and control plants was analyzed (Table S2 and Figure 2). When compared to control, in TYLCTHV-infected plants 2322 and 1377 genes were upregulated and downregulated, respectively (Figure 2). Functional annotation using the Kyoto Encyclopedia of Genes and Genomes (KEGG) database sorted DEGs into 20 unique pathway groups, spanning across five major pathway categories (Figure S3). The highest numbers of DEGs were observed in the pathway Carbohydrate metabolism and Energy metabolism within the Metabolism pathways; in contrast, only several dozens of DEGs were identified in the categories Cellular Processes and Organismal Systems (Figure S3).

### 2.4. Functional Enrichment of DEGs

**Figure 3:**
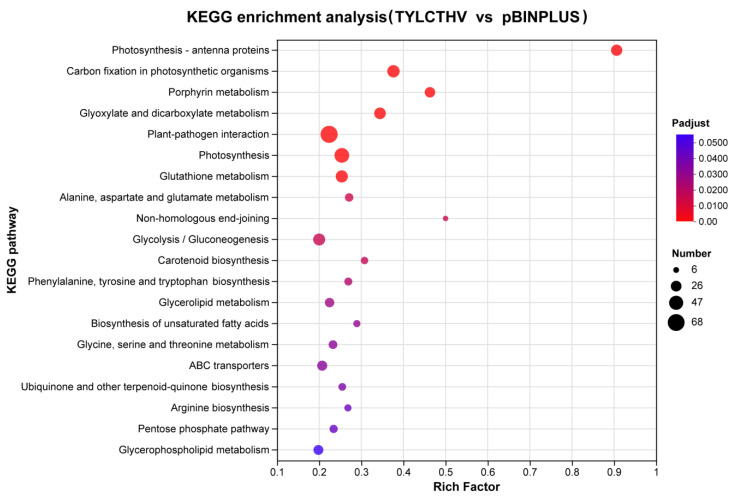
Distribution of the top 20 most significantly enriched KEGG pathways. The Y-axis represents the identity of pathway, and the X-axis indicates the rich factor. The adjusted *p* value is indicated by the color of the dots. The number of DEGs involved in each pathway is represented by the size of the dots.

To identify the biological processes regulated by DEGs, KEGG enrichment analysis was conducted. Various pathways were identified (Table S3), of which the most significantly enriched were presented (Figure 3). Many pathways associated with plant photosynthesis processes were among the most significantly enriched, including Photosynthesis—antenna proteins, Carbon fixation in photosynthetic organisms and Photosynthesis. Following these were some pathways involved in carbohydrate metabolism including Glyoxylate and dicarboxylate metabolism and Glycolysis/Gluconeogenesis. The fifth most significantly enriched pathway was Plant-pathogen interaction. Also enriched were several amino acid biosynthesis pathways including Glutathione metabolism and Alanine, aspartate and glutamate metabolism, among others.

### 2.5. Qrt-PCR Verification of Transcriptomic Data

**Figure 4:**
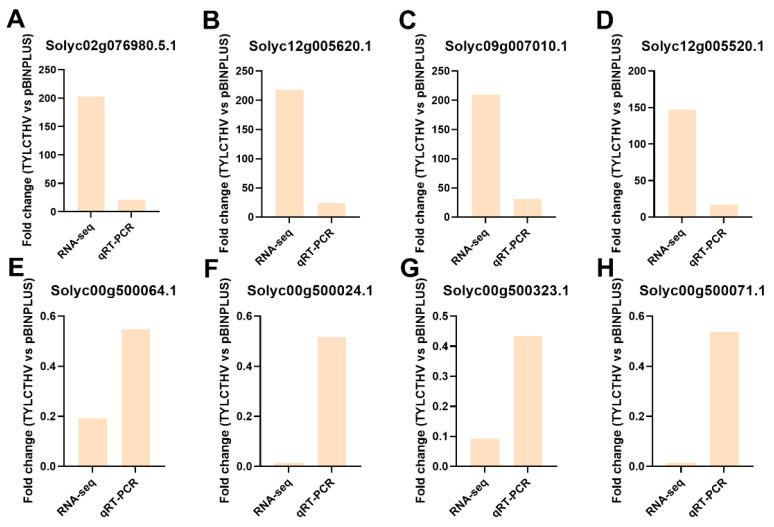
qRT-PCR validation of the fold change (TYLCTHV vs. pBINPLUS) of DEGs. (**A**–**D**) DEGs in the pathway Photosynthesis; (**E**–**H**) DEGs in the pathway Plant-pathogen interaction. The number of replicates was 4 for RNA-seq and 5–6 for qRT-PCR.

qRT-PCR was conducted to verify the expression of DEGs. We selected four DEGs that were of the highest fold change value from the pathway Plant-pathogen interaction and four DEGs that were of the lowest fold change value from the pathway Photosynthesis. The two pathways were chosen because the expression of genes in the two pathways was constantly modulated in plants upon begomovirus infection [19,20,21]. The transcript levels of the four selected DEGs from the pathway Plant-pathogen interaction (Solyc02g076980.5.1, Solyc12g005620.1, Solyc09g007010.1 and Solyc12g005520.1) were upregulated (fold change > 1) in RNA-seq data. Similarly, in qRT-PCR assay their expression was all upregulated (fold change > 1), verifying the RNA-seq data (Figure 4A–D). The regulation of four DEGs in the pathway Photosynthesis (Solyc00g500064.1, Solyc00g500024.1, Solyc00g500323.1 and Solyc00g500071.1) was also consistent in RNA-seq and qRT-PCR (fold change < 1) (Figure 4E–H). These data suggest that the regulation of the expression of the DEGs found in the RNA-seq data were reliable.

### 2.6. Interference of Plant Photosynthesis Processes by TYLCTHV

**Figure 5:**
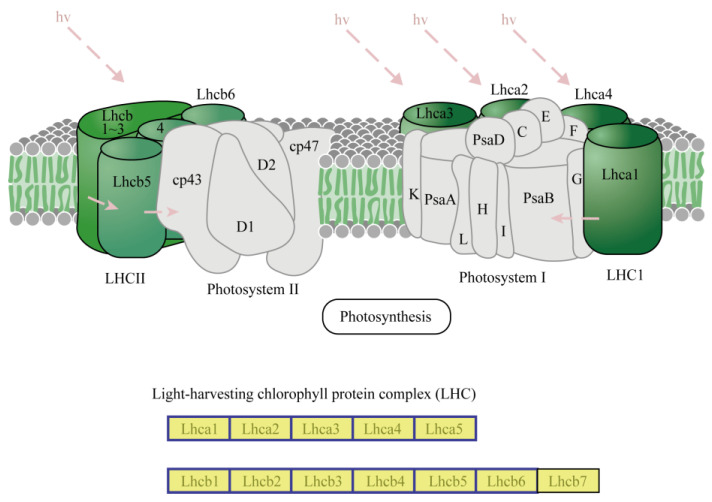
Regulation of the expression of DEGs in the pathway Photosynthesis - antenna proteins. The major components in this pathway are shown. Blue and red frames signify downregulation and upregulation of gene expression, respectively. Green fill-ins indicate newly identified transcripts, and yellow fill-ins indicate previously identified transcripts.

Three pathways were closely related to plant photosynthesis processes, including Photosynthesis-antenna proteins, Carbon fixation in photosynthetic organisms and Photosynthesis. We thus pay specific attention to these pathways. Photosynthesis-antenna proteins was the top most significantly enriched KEGG pathway. The rich factor and adjusted *p* value for this pathway were 0.90625 and 2.45 × 10^−22^, respectively (Table S3). The expression of all the 29 DEGs in this pathway was downregulated, and the fold changes for these DEGs were 0.155–0.442 (Table S4). Examination of these DEGs revealed that most components (11/12) of the plant light-harvesting chlorophyll protein complex, including LHca1-5 and LHcb1-6, were negatively modulated by TYLCTHV (Figure 5).

**Figure 6:**
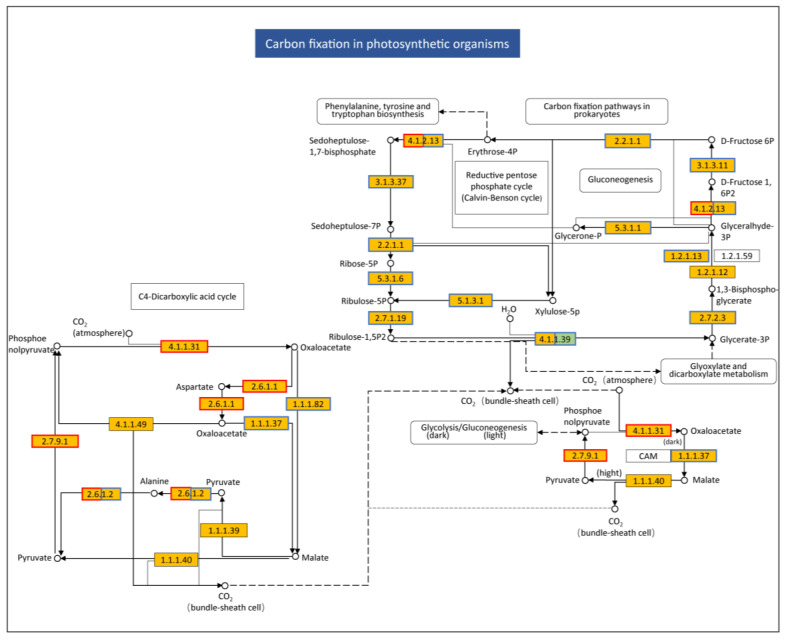
Changes in the expression of DEGs in the pathway Carbon fixation in photosynthetic organisms induced by TYLCTHV infection. Chemical conversion is shown with the catalytic enzymes involved. Blue and red coloring of the frames denote expression downregulation and upregulation, respectively. Boxes with green fill-ins show newly identified transcripts, and yellow boxes with fill-ins indicate known transcripts.

Carbon fixation in photosynthetic organisms is the second most significantly enriched pathway, and its rich factor and adjusted *p* value were 0.376 and 4.13 × 10^−9^, respectively (Table S3). The expression of the majority (29/35) of DEGs was downregulated, and the expression of six DEGs was upregulated. The fold changes were 0.16–0.47 for downregulated DEGs and 2.18–5.72 for upregulated DEGs, respectively (Table S5). Examination of DEGs showed that the expression of many DEGs encoding enzymes involved in carbon fixation was downregulated (Figure 6). For example, the expression of DEGs encoding E3.1.3.37, E2.2.1.1, E5.3.1.6 and E2.7.1.19, which are responsible for the conversion from sedoheptulose-1,7-bisphophate to ribulose-1,5P_2_, was downregulated. Intriguingly, the expression of some DEGs regulating the C4-dicarboxylic acid cycle was upregulated, such as E2.7.9.1, which is responsible for the conversion from pyruvate to phosphoenolpyruvate, and E4.1.1.31, which is responsible for the conversion from phosphoenolpyruvate to oxaloacetate.

**Figure 7:**
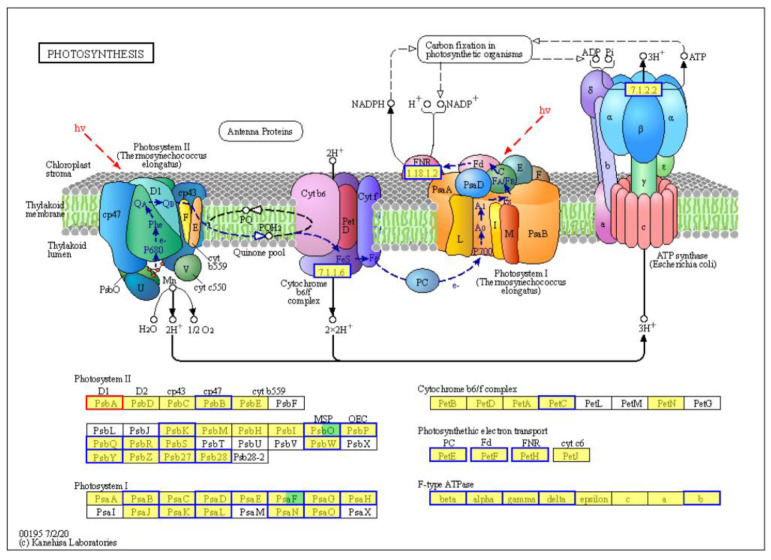
Regulation of the expression of DEGs in the pathway Photosynthesis. Blue and red frames denote downregulation and upregulation, respectively. Green and yellow fill-ins indicate newly identified and known transcripts, respectively.

Photosynthesis ranked sixth in the top enriched pathways (rich factor: 0.25, adjusted *p* value: 1.23 × 10^−6^ (Table S3). In this pathway, downregulation of the expression of 49 DEGs was found, and only one DEG was upregulated (Table S6). The 49 DEGs were downregulated by 54.5–98.5% upon TYLCTHV infection. Notably, TYLCTHV affected all the components of the photosynthesis complex (Figure 7). Specifically, downregulation was found for *PsbB*, *PsbK*, *PsbO*, *PsbP*, *PsbQ*, *PsbR*, *PsbS*, *PsbW*, *PsbY*, *Psb27* and *Psb28* in photosystem II. The expression of the homologs of *PsaB*, *PsaD*, *PsaE*, *PsaF*, *PsaG*, *PsaH*, *PsaK*, *PsaL*, *PsaN* and *PsaO* in photosystem I were downregulated. Similarly, *PetC* (cytochrome b6/f complex) homolog and *PetE*, *PetF* and *PetH* (involved in photosynthetic electron transport) were downregulated. Also downregulated were the expressions of *F-type ATPase beta*, *alpha, gamma*, *delta* and *b*.

### 2.7. Induction of Plant Defense Responses by TYLCTHV

**Figure 8:**
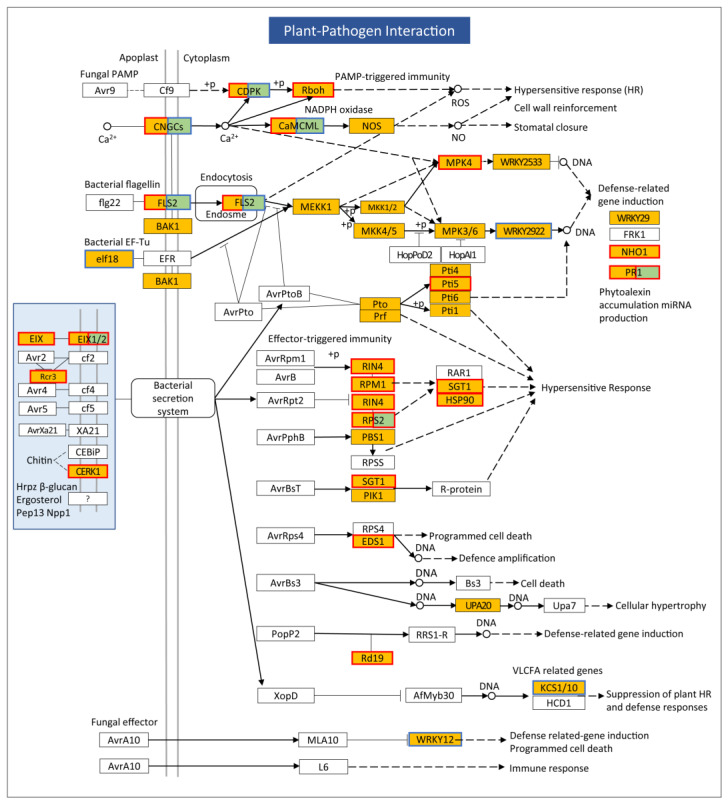
Regulation of the expression of DEGs in the pathway Plant-pathogen interaction. The major components regulating plant–pathogen interaction are shown. Blue and red frames signify downregulation and upregulation of gene expression, respectively. Green fill-ins indicate newly identified transcripts, and yellow fill-ins indicate previously identified transcripts.

Plant-pathogen interaction ranked fifth in the KEGG enriched analysis (rich factor: 0.22, adjusted *p* value: 1.07 × 10^−6^) (Table S3). Most (57) of the 68 DEGs in this pathway were upregulated (Table S7). The expression of upregulated DEGs increased by 100–21,641% upon TYLCTHV infection. The expressions of 11 downregulated DEGs were reduced by 51.83–72.61%. Specifically, TYLCTHV modulated many plant defense pathways (Figure 8). For example, the expression of many DEGs regulating pathogen-associated molecular pattern (PAMP)-triggered immunity increased, such as *EIX*, *EIX1/2*, *Rcr3*, *CERK1*, *CDPK*, *Rboh*, *CNGCs CaM/CML* and *FLS2*. Similar upregulation was found for the expression of *MPK4*, *Pti5*, *NHO1* and *PR1*, which regulate defense-related gene induction. The expression of *RIN4*, *RPM1*, *TPS2*, *PBS1*, *SGT1*, *EDS1* and *HSP90*, which regulate effector-triggered immunity, was induced by TYLCTHV infection.

## 3. Discussion

While how tomato interacts with monopartite begomoviruses during the pathogenesis of TYLCD has been examined extensively, tomato interactions with bipartite begomoviruses remain understudied. To address this knowledge gap, here we explored the transcriptomic changes in tomato plants in response to infection with a bipartite begomovirus, TYLCTHV. We first characterized changes in tomato plant phenotype in response to TYLCTHV and further verified virus infection status in pBINPLUS- and TYLCTHV-infected plants with PCR. Virus infection in tomato induced typical symptoms of TYLCD including stunted growth, leaf curl and yellowing. We next profiled tomato transcriptomic changes upon TYLCTHV infection and identified 2322 upregulated and 1377 downregulated DEGs. Functional enrichment analysis revealed that many KEGG pathways involved in plant photosynthesis and defense responses were enriched. Detailed examination of DEGs in these pathways showed that TYLCTHV actively disrupts plant photosynthesis processes and induces defense responses in tomato plants.

In plants, photosynthesis begins with light absorption by the light-harvesting chlorophyll protein complex (antenna proteins) of the photosystems, followed by excitation energy transfer to the reaction centers; next, electron and proton transport are followed NADPH and ATP synthesis being initiated and, finally, fixation of CO_2_ occurs [22]. Here we found that upon TYLCTHV infection, all the major apparatuses of photosynthesis were disrupted, including the light-harvesting chlorophyll protein complex, photosystem I and II, cytochrome b6/f complex, photosynthetic electron transport and F-type ATPase. Alternations in the function of these apparatuses may be responsible for the genesis of TYLCD symptoms including stunted growth and leaf yellowing due to reduced light energy assimilation and chlorophyll protein availability. The disruption of plant photosynthesis processes has also been documented in the interaction between tomato and the monopartite begomoviruses that cause TYLCD. For example, upon the TYLCV infection of tomato plants, the expression of many DEGs encoding regulators of light reaction, photo respiration and the Calvin cycle of photosynthesis was downregulated [21]. Further exploration of the molecular mechanisms underlying the manipulation of plant photosynthetic apparatuses by TYLCD-causing begomoviruses will help to dissect disease pathogenesis, thereby offering novel targets for symptom attenuation.

Plants defend themselves against invading pathogens by mounting a repertoire of defense responses including PAMP-triggered immunity and effector-triggered immunity [23]. In addition, phytohormones such as salicylic acid also play a role in conferring resistance against viral pathogens [24]. In this study, we found that the expression of many DEGs regulating plant defense mechanisms including PAMP-triggered immunity, effector-triggered immunity and hypersensitive response was upregulated. Additionally, dramatic upregulation of the expression of *pathogenesis-related protein 1* (*PR1*) was found in response to TYLCTHV infection. *PR1* is a downstream gene of the salicylic acid signaling pathway that is often used as the indicator of the activation status of this pathway [25]. The upregulation of these DEGs indicates the activation of multiple plant immune networks. It should be noted, however, the activation of plant immunity did not seem to inhibit TYLCTHV pathogenesis as obvious virus-induced symptoms were present. Under this scenario, further studies may focus on the induction and evasion of plant defenses by TYLCTHV, thereby providing reference for the improvement of plant resistance against TYLCD.

## 4. Materials and Methods

### 4.1. Plants and Virus

Tomato (*Solanum lycopersicum* L. cv. Hezuo903) plants were grown in insect-proof greenhouses under natural lighting. Tomato yellow leaf curl Thailand virus (TYLCTHV) isolate Guangdong (DNA-A GenBank accession no.: OM912581, DNA-B GenBank accession no: OM912582) was used. Infectious clones were kindly provided by Dr. Yafei Tang (Plant Protection Research Institute, Guangdong Academy of Agricultural Sciences). Agrobacteria containing infectious clones of DNA-A and DNA-B were cultured separately until the OD_600_ reached 2.0, then pelleted and resuspended in resuspension buffer (10 mM MgCl_2_, 10 mM MES, 200 µM acetosyringone). Equal quantities of agrobacteria containing infectious clones of DNA-A and DNA-B were mixed and used for inoculation. To obtain virus-infected plants, agrobacteria were introduced into tomato plants that were at 2–3 true-leaf stage (about 4 weeks post sowing) using 1 mL syringes. Plants inoculated with agrobacteria containing pBINPLUS (empty vector) were used as un-infected controls. Three weeks post-agro-inoculation, plants were sampled and DNAs were extracted with the Plant Genomic DNA Kit (Tiangen, Beijing, China). TYLCTHV DNA-A was detected with PCR using the primers listed in Table S1, and the plants were then used for transcriptomic analysis.

### 4.2. CDNA Library Preparation and Illumina Sequencing 

The second and third fully expanded leaves of tomato plants were harvested, and leaves from three plants were mixed as one sample. Sample were collected in quadruplicate for both TYLCHV and control treatments to ensure biological reliability. cDNA library preparation and high-throughput sequencing were conducted by Majorbio Bio-Pharm Technology Co., Ltd. (Shanghai, China), as per the manufacturer’s protocols. Total RNAs were extracted using TRIzol (Ambion, Austin, TX, USA), followed by an RNA quality assessment using a 5300 Bioanalyser (Agilent, Santa Clara, CA, USA) and quantification with an ND-2000 (NanoDrop, Waltham, MA, USA). Only high-quality RNA samples (OD_260/280_ = 1.8~2.2, OD_260/230_ ≥ 2.0, RQN ≥ 6.5, 28S:18S ≥ 1.0, >1 μg) were used to construct the sequencing library. The libraries were prepared using the Illumina Stranded mRNA Prep and Ligation method, starting with 1 μg of total RNA. The workflow included isolating messenger RNA through polyA selection using Oligo (dT) beads (Invitrogen, Carlsbad, CA, USA), followed by fragmentation using a fragmentation buffer. Subsequently, double-stranded cDNA was synthesized using the SuperScript double-stranded cDNA synthesis kit (Invitrogen, Carlsbad, CA, USA), with random hexamer primers. The cDNA was then processed for end-repair, phosphorylation and adapter ligation as per the library construction protocol. The libraries were size-selected to target cDNA fragments of 300 bp using a 2% Low Range Ultra Agarose gel, followed by PCR amplification with Phusion DNA polymerase (New England Biolabs, Beverly, MA, USA) for 15 cycles. After quantification with the Qubit 4.0 fluorometer, the sequencing library was sequenced on the NovaSeq X Plus platform (PE150) using the NovaSeq Reagent Kit (Novagen, Madison, WI, USA).

### 4.3. Quality Control and Read Mapping

To eliminate sequencing adapters, low-quality reads, sequences with a high incidence of bases with uncertain information and excessively short sequences within the original sequencing data, the raw paired-end reads were subjected to trimming and quality control using the software fastp with its default parameters [26]. Upon the completion of data quality control, re-evaluation of data quality was conducted. This reassessment encompassed a statistical analysis of the distribution of base error rates and the distribution of base content. Clean reads were then separately aligned to the tomato reference genome (*Solanum lycopersicum* SL4, https://www.solgenomics.net/organism/Solanum_lycopersicum/genome/, accessed on 8 October 2024) [27] with orientation mode using HISAT2 2.1.0 [28]. The alignment results of transcriptome sequencing are also evaluated with HISAT2, including the saturation of sequencing, gene coverage, distribution of reads in different regions of the reference genome and distribution of reads on different chromosomes. The mapped reads of each sample were assembled with StringTie in a reference-based approach [29].

### 4.4. Transcript Assembly and Analysis of Gene Expression Level

On the basis of the *Solanum_lycopersicum* reference genome (SL4.0, https://www.solgenomics.net/organism/Solanum_lycopersicum/genome/, accessed on 8 October 2024) [27], the mapped reads are assembled using the software Cufflinks 2.2.1 or StringTie 2.1.2 [29,30] and compared with the known transcripts to obtain transcripts with annotation information and potential new transcripts. The level of gene expression is indicated by the abundance of transcripts. In RNA-seq data analysis, the expression level of genes is calculated based on the number of sequences (clean reads) that align to genomic regions, known as read counts. The software RSEM 1.3.3 is utilized to quantify gene expression levels, facilitating subsequent analyses to identify the differential gene expression between different samples [31]. After obtaining the read counts for genes, differential expression analysis between the treatments was performed to identify differentially expressed genes (DEGs). The software used for differential expression analysis was DESeq2 1.24.0 [32], with the default criteria for significant differential expression being: FDR (False Discovery Rate) < 0.05 and |log_2_FC (log_2_ Fold Change)| ≥ 1. A gene is considered a DEG when it meets both criteria.

### 4.5. Functional Enrichment Analysis of DEGs

Functional enrichment analysis of DEGs was conducted to identify overrepresented biological processes, molecular functions and cellular components, as well as to uncover the significant pathways involved. The KEGG pathway enrichment analysis was performed using R-script to identify pathways that were significantly associated with the DEGs [33]. The analysis was based on the Fisher’s exact test, with a threshold of Padjust <0.05 to denote statistical significance for enriched KEGG terms.

### 4.6. Quantitative Reverse-Transcription PCR (qRT-PCR)

Samples for qRT-PCR were harvested from the second and third fully expanded leaves of tomato plants, and leaves from three plants were mixed as one sample. To analyze the gene expression level using qRT-PCR, total RNAs were extracted from plant samples using TRIzol and reverse-transcribed using an Evo M-MLV RT Kit with gDNA Clean for qPCR (Accurate Biology, Shanghai, China). Quantitative PCR was performed using a SYBR Green Premix Pro Taq HS qPCR Kit (Accurate Biology, Shanghai, China) and the CFX96 Real Time PCR Detection System (Bio-Rad, Hercules, CA, USA), with the primers listed in Table S1. *β-Tubulin* (gene ID: Solyc04g081490.3) was used as a reference gene in the qRT-PCR analysis of gene expression as its expression was stable between control and TYLCTHV-infected plants. Each sample was tested using two technical replicates. The reaction protocol was as follows: initial denaturation at 95 °C (30 s), denaturation at 95 °C (5 s), annealing and extension at 60 °C (34 s), for a total of 40 cycles, with a melting curve included to assess primer specificity. Finally, the gene expression levels were calculated using the 2^−ΔΔCt^ method as normalized to *β-tubulin*.

## 5. Conclusions

Taken together, here we found that TYLCTHV infection induces typical TYLCD symptoms in tomato plants. Furthermore, TYLCTHV disrupts multiple steps of plant photosynthesis processes and induces an array of plant defense responses. Our findings add to our knowledge of TYLCD pathogenesis and plant–virus interactions in general.

## Figures and Tables

**Table 1:** Sequencing data statistics.

Sample	Raw Reads	Clean Reads	Error%	Q20%	Q30%	GC%
pBINPLUS_1	49,891,168	49,476,800	0.0123	98.57	95.56	43
pBINPLUS_2	64,534,292	63,907,418	0.0123	98.57	95.53	43.42
pBINPLUS_3	54,661,946	54,296,048	0.0122	98.59	95.6	43.11
pBINPLUS_4	48,012,992	47,627,718	0.0124	98.52	95.38	42.43
TYLCTHV_1	56,556,462	56,007,018	0.0123	98.54	95.45	41.75
TYLCTHV_2	58,147,208	57,713,032	0.0122	98.59	95.61	41.64
TYLCTHV_3	43,688,846	43,341,960	0.0123	98.55	95.47	42.17
TYLCTHV_4	49,327,424	48,968,828	0.0123	98.57	95.53	42.35

**Table 2:** Mapping of clean reads to the reference genome.

Sample	Total Reads	Total Mapped	Multiple Mapped	Uniquely Mapped
pBINPLUS_1	49,476,800	48,600,635(98.23%)	1,889,678(3.82%)	46,710,957(94.41%)
pBINPLUS_2	63,907,418	62,747,944(98.19%)	2,603,437(4.07%)	60,144,507(94.11%)
pBINPLUS_3	54,296,048	53,260,364(98.09%)	1,922,290(3.54%)	51,338,074(94.55%)
pBINPLUS_4	47,627,718	46,783,258(98.23%)	1,539,095(3.23%)	45,244,163(95.00%)
TYLCTHV_1	56,007,018	53,991,001(96.40%)	1,560,371(2.79%)	52,430,630(93.61%)
TYLCTHV_2	57,713,032	54,568,886(94.55%)	2,173,070(3.77%)	52,395,816(90.79%)
TYLCTHV_3	43,341,960	41,758,846(96.35%)	1,147,672(2.65%)	40,611,174(93.70%)
TYLCTHV_4	48,968,828	47,174,688(96.34%)	1,473,859(3.01%)	45,700,829(93.33%)

## Data Availability

The raw data from RNA-seq were deposited in the Sequence Read Archive database under the project number PRJNA1134623.

## Supplementary Materials

The following supporting information can be downloaded at: https://www.mdpi.com/article/10.3390/plants13223198/s1. Figure S1. PCR detection of TYLCTHV DNA-A in pBINPLUS-inoculated and TYLCTHV-inoculated tomato plants; Figure S2. Length distribution of transcripts; Figure S3. KEGG functional classification of DEGs; Table S1. Primers used in this study; Table S2. Expression level of all transcripts; Table S3. Summary of KEGG enrichment analysis; Table S4. Expression of DEGs in the pathway Photosynthesis - antenna proteins; Table S5. Expression of DEGs in the pathway Carbon fixation in photosynthetic organisms; Table S6. Expression of DEGs in the pathway Photosynthesis; Table S7. Expression of DEGs in the pathway Plant-pathogen interaction.
