# Supplementary material for: Transcriptomic Profiling Unravels the Disruption of Photosynthesis Apparatuses and Induction of Immune Responses by a Bipartite Begomovirus in Tomato Plants
Source: Plants (Basel). 2024 Nov 14;13(22):3198. doi: 10.3390/plants13223198 (PMC11598137; doi:10.3390/plants13223198)
Supplement: Supplementary file 1 [file plants-13-03198-s001.zip › Figure S1-S3 1031.pdf]

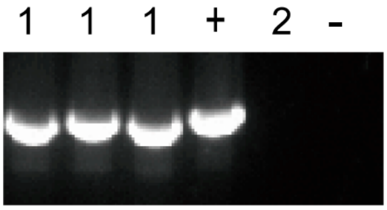

Fig. S1 PCR detection of TYLCTHV DNA-A in pBINPLUS-inoculated and TYLCTHV-inoculated tomato plants. At three weeks post inoculation, the first fully-expanded leaves were harvested and subjected to the detection of TYLCTHV DNA-A. + and – stand for positive (agrobacteria) and negative (water) control, respectively. 1 is TYLCTHV DNA-A+DNA-B-inoculated plants, and 2 is pBINPLUS-inoculated plants.

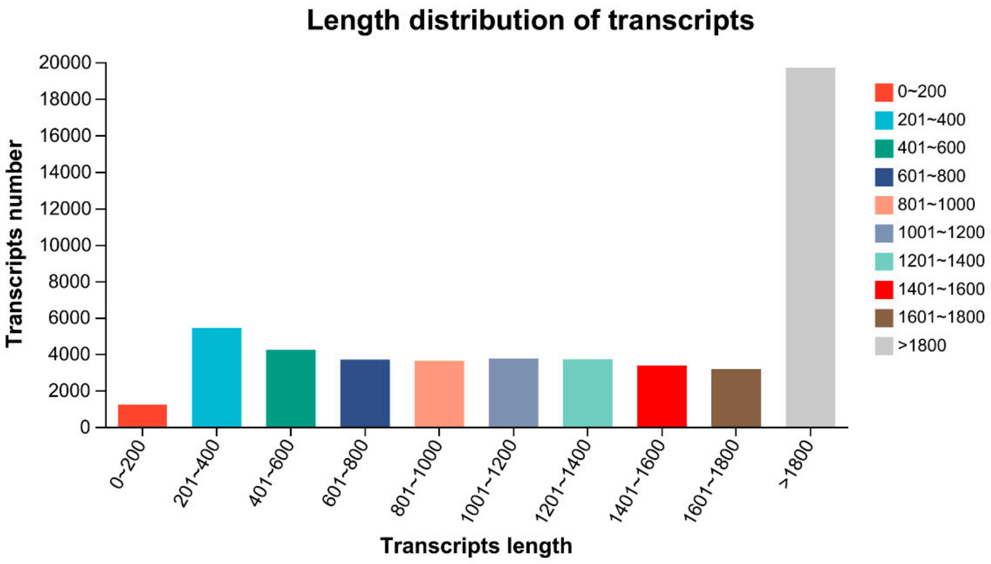

Fig. S2 Length distribution of transcripts.

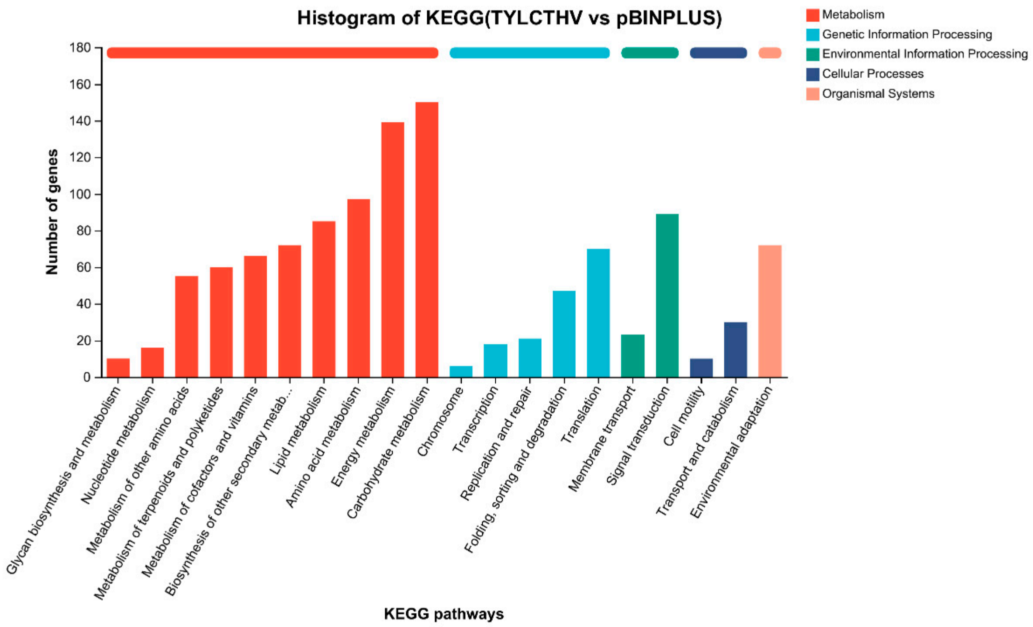

Fig. S3 KEGG functional classification of DEGs.
